# Development of mechano-responsive polymeric scaffolds using functionalized silica nano-fillers for the control of cellular functions

**DOI:** 10.1016/j.nano.2016.02.011

**Published:** 2016-08

**Authors:** Michelle Griffin, Leila Nayyer, Peter E. Butler, Robert G. Palgrave, Alexander M. Seifalian, Deepak M. Kalaskar

**Affiliations:** a UCL Centre for Nanotechnology and Regenerative Medicine, Division of Surgery & Interventional Science, University College London, London, United Kingdom; b Royal Free London NHS Foundation Trust Hospital, London, United Kingdom; c Department of Chemistry, University College London, London, United Kingdom

**Keywords:** Fumed silica, Nanoparticles, Polyhedral oligomeric silsesquioxane, Hydroxyl and amine functionalization, Mechano-responsive scaffolds

## Abstract

We demonstrate an efficient method to produce mechano-responsive polymeric scaffolds which can alter cellular functions using two different functionalized (OH and NH_2_) silica nano-fillers. Fumed silica-hydroxyl and fumed silica-amine nano-fillers were mixed with a biocompatible polymer (POSS-PCU) at various wt% to produce scaffolds. XPS and mechanical testing demonstrate that bulk mechanical properties are modified without changing the scaffold's surface chemistry. Mechanical testing showed significant change in bulk properties of POSS-PCU scaffolds with an addition of silica nanofillers as low as 1% (*P* < 0.01). Scaffolds modified with NH_2_ silica showed significantly higher bulk mechanical properties compared to the one modified with the OH group. Enhanced cell adhesion, proliferation and collagen production over 14 days were observed on scaffolds with higher bulk mechanical properties (NH_2_) compared to those with lower ones (unmodified and OH modified) (*P* < 0.05) during *in vitro* analysis. This study provides an effective method of manufacturing mechano-responsive polymeric scaffolds, which can help to customize cellular responses for biomaterial applications.

## Graphical Abstract

Incorporation of functional nano-silica fillers modifies bulk mechanical properties of the POSS-PCU nanocomposite scaffold. By altering the type of functional silica used, mechanical properties can be tailored to either encourage or discourage cellular responses. This strategy can help to customize biological implants.

**Figure:**
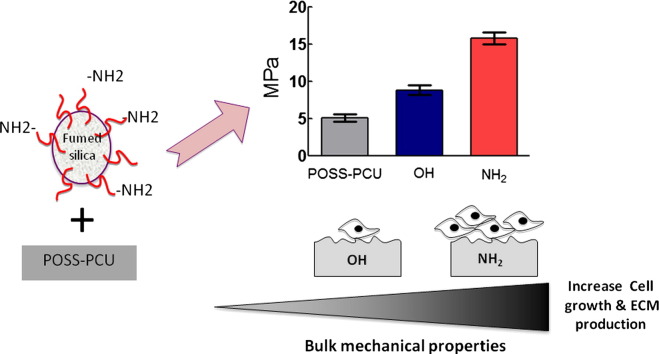

Many cell types have shown to alter their morphology and gene profile when grown on chemically equivalent surfaces with different rigidities. Previous studies have shown that the substrate stiffness can regulate cellular behavior in terms of adhesion, proliferation, migration and differentiation.^1^, ^2^ To understand the cell-substrate interphase the elastic modulus for different extracellular matrices has been characterized, for example bone tissue is around 15 MPa and skeletal tissue is approximately 10 kPa.^3^ Various studies have shown that different cell types prefer stiffer materials with increased cytoskeletal development and adhesion on stiffer materials.^4^ Hopp et al^4^ found that fibroblast adhesion, proliferation and morphology were affected by substrate elasticity. Human dermal fibroblasts were found to attach and proliferate on the stiffer regions of the substrate.^4^ Cell adhesion, cell cycle progression and ultimately proliferation are often dependent on the cell-adhesive proteins on the surface of materials.^5^ Cell adhesion to stiffer surfaces has been accounted for by increased adsorption and optimal ordering/disordering state of the adsorbed proteins.^5^, ^6^, ^7^ Hence, consideration of substance rigidity has become an essential aspect of biomaterial science while designing new implant interphases.

Current methods to increase the stiffness of polymers include varying the structure of the polymer including the molecular weight, crosslinking and degree of steric order of the main polymer chain.^8^ However, simple modifications without changing the polymer structure include the addition of nanoparticles as bulk fillers.

Extensive amount of research has been focused on using silica nanoparticles for biomedical applications including biological imaging, biosensors and tissue engineering due to their large surface area, low toxicity and tunable chemical structure.^9^, ^10^ However, only a few studies have investigated the use of nano-silica particles for modification of polymers for biomaterial applications. Schiraldi and colleagues^11^ investigated the use of fumes silica particles to produce hybrid hydrogels with poly(hydroxyethylmethacrylate) (pHEMA). They reported improved cell adhesion and proliferation of 3T3-fibroblast cells when silica nanoparticles were used at 10% and 30% concentrations.^11^ A follow-up study by D'Agostino and colleagues^12^ showed improved dispersion of fume silica with pHEMA hydrogel by functionalizing it with using methacryloylpropltrimetoxy silane (MPTMS). Gaharwar et al^13^ demonstrated that PEG hydrogels enhanced initial cell adhesion, promoted cell spreading and increased the metabolic activity of the cells after the addition of silica nanospheres. A recent study by our group showed functionalization of polymeric scaffolds by fumed silica particles tethered with RGD peptides to capture endothelial cells for vascular bypass graft applications.^10^ However to date, no study has reported the effect of mechanical properties due to the incorporation of silica nano-particles into polymeric scaffolds on cellular behavior.

Current study presents a simple and efficient method to produce mechanically varied polymeric scaffolds using two different nano-silica fillers. The effect of these scaffolds on controlling cellular behavior is also reported in detail. In this study, we used a nanocomposite polymer called polyhedral oligomeric silsesquioxanes-poly(carbonate-urea) urethane (POSS-PCU). This nanocomposite material is a novel biocompatible and non-toxic polymer. It has been used in patients as the world's first synthetic adult trachea, lachrymal duct and lower limb bypass.^14^, ^15^

Nanosilica particles with inherent hydroxyl functionality (–OH) were modified via amino-silanization to introduce amine (–NH_2_) functionality. Two types of fume silica nano-particles produced with (–OH) and (–NH_2_) functionalities were then mixed with a biocompatible polymer (POSS-PCU) to produce scaffolds with different mechanical properties. Surface and mechanical characterization of scaffold was carried out using X-ray photon spectroscopy (XPS), atomic force microscopy (AFM), scanning electron microscopy (SEM) and instron mechanical tester. Results showed that scaffolds with varied mechanical properties can be produced using fumed silica particles, without affecting the surface chemistry.

In order to evaluate if a functional silica filler incorporated into a scaffold provides any advantage for biomaterials applications over unmodified samples, human dermal fibroblast cell behavior was evaluated in *in vitro* cell culture using various fluorescent and biochemical assays such as Alamar blue, DNA, collagen production, histology and cytoskeleton staining. Results demonstrated that the scaffolds with a higher bulk mechanical properties enhance fibroblast cellular functions compared to scaffolds with lower mechanical properties. This study thus provides a simple but efficient method to control cellular functions by controlling mechanical properties of scaffolds.

## Methods

### Nanocomposite polymer synthesis

The nanocomposite polymer was synthesized, as described previously.^16^ Briefly, polycarbonate polyol (2000 Mwt) and *trans*-cyclohexanechloroydrinisobutyl-silses-106 quioxane (Hybrid Plastics Inc) were placed into a 500 ml flask containing a mechanical stirrer and under nitrogen environment. The POSS cages were dissolved into the polyol solution at 70 °C. Flakes of 4,4′-methylenebis(phenyl 109 isocyanate) (MDI) was added to the polyol blend mixture between 80 and 90 °C to form a pre-polymer. To create a polymer solution, dimethylacetamide (DMAC) was then added slowly to the pre-polymer. Chain extension was carried out by cooling solution to 40 °C, followed by the addition of ethylenediamine and diethylamine in DMAC in a dropwise manner. This process leads to the synthesis of the POSS-modified polycarbonate urea–urethane in DMAC solution.

### Functionalization of fume silica

NH_2_ functionalization of fume silica was done by silanization process. 1 g of fumed silica (AEROSIL® 200, evonik industries, Germany) along with 100 ml iso-propanol (IPS) was added to the refluxing flask. The mixture was sonicated for 30 min. 1 ml of aminopropyltrimethoxysilane (APS) and 1 ml of H_2_O were introduced into the reaction flask. The resulting solution then underwent reflux for 30 min at 80 °C. The particles were then filtered and washed with IPS thoroughly.

### Nanocomposite scaffold fabrication

The POSS-PCU based scaffold was manufactured using a salt leaching method as described previously.^8^ Briefly, sodium chloride (NaCl) of known size (150-250 μm) was added in 18% wt solution of POSS-PCU in DMAC containing Tween-20 surfactant. The final solution was then dispersed and degassed in a Thinky AER 250 mixer (Intertonics, Kidlington, UK). A 3:7 weight ratio of NaCl to POSS-PCU was used in all experiments. When using the solvent casting technique, the final mixture was spread evenly onto a circular steel mould (8 cm × 8 cm) and left in an oven at 65 °C for 4-5 h until all the solvents had evaporated. This procedure was repeated twice to ensure that the polymer was of an adequate thickness. Casted polymer was then removed from modules and placed in deionized water for 7 days to remove all NaCl which produce porous scaffolds. Water was changed twice daily. For cell culture analysis, samples were cut into circular discs of 16 mm diameter to put in 24-well plates, using a stainless steel cutter.

Fumed silica (OH) and fumed amine (NH_2_) modified POSS-PCU scaffolds were prepared as per process described below. Both OH and NH_2_ fumed silicas were added to the polymetic solution of POSS-PCU (18 wt%) in DMAC along with Tween-20 and sonicated for 30 min to ensure adequate mixing. Scaffolds of variable mechanical properties were manufactured by incorporating both fume silicas at 1, 2 and 4 (wt%) with POSS-PCU polymeric solution. Scaffolds were manufactured using salt leaching method as per the process described above. Scaffolds fabricated following incorporation of fumed (OH) and fumed amine (NH_2_) were referred to as OH and NH_2_ throughout the manuscript.

#### Polymer characterization

To characterize the OH and NH_2_ functionalized scaffolds the following techniques were implemented:

##### Mechanical testing

The mechanical properties of POSS-PCU modified and unmodified (OH, NH_2_) were characterized using the Instron-5565 tensile 224 tester as previously described.^16^ Briefly, samples were cut into standard dog bone shape (20 × 4 mm) and tensile properties were analyzed at a loading speed of 50 mm/min. The scaffold thickness was measured using an Vernear Caliper. Average Young's modulus of elasticity was reported following analysis of six independent samples (n = 6).

##### Scanning electron microscope (SEM)

Scaffold morphology, pore size and their distribution were analyzed using SEM. To study cell materials interactions, cell seeded scaffolds were fixed with 2.5% w/v glutaraldehyde/PBS for 1 h.^16^ The scaffolds were then dehydrated using a series of alcohol solutions at room temperature and then allowed to air dry. The polymer disk scaffolds were then attached to aluminium stubs with double-sided sticky tabs before being coated with gold using an sc500 (EMScope) sputter coater. The scaffolds were then photographed and analyzed using the Carl Zeiss's Advanced Analytical Scanning Electron Microscope (SEM), with the SMART software package (n = 3).

##### X-ray photoelectron spectroscopy (XPS) analysis

The surface composition and chemistry of the modified scaffolds were characterized using a Thermo Scientific K-alpha spectrometer (Department of Chemistry, UCL, UK). Monochromatic Al Kα X-rays (*h* = 1486.6 eV) were focused to a 400 μm diameter spot on the sample surface, defining the analysis area. The analysis depth at this photon energies was 5-10 nm. Survey spectra were conducted to determine the elemental composition of the surface. High-resolution spectra of the principle core line of each element present were then acquired for chemical state identification. Where necessary, these high resolution spectra were fitted with Gaussian–Lorentzian peaks using CasaXPS software to deconvolute different chemical environments.

##### Atomic force microscopy (AFM)

The POSS-PCU surface topography was examined using an atomic force microscope (TAP150A) operating in tapping mode using a spring constant 2.919 n/m. The root mean square roughness (RSM) was calculated from the 5 μm scan of three areas using the NanoScope® analysis software (Bruker Corporation) version 1.40 (n = 3).

##### Transmission electron microscopy (TEM)

To assess the size of the fumed silica nanoparticles TEM was utilized. The fumed silica and fumed silica functionalized with amine nanoparticles were dropped on copper grids and imaged using a JEOL JEM-1010 and captured using a Gatan CCD Camera.

#### Cell culture and cell seeding

Human dermal fibroblasts (HDF) primary cells obtained from the European Collection of Cell Culture (ECACC) were cultured in Dulbecco's Modified Eagle's medium (DMEM) supplemented with 10% fetal bovine serum (FBS) and 1% antibiotic (50 U/ml penicillin) solutions (all from Sigma, UK). For cell culture experiments the 16 mm polymer scaffolds were autoclaved and washed with sterile phosphate buffer solution (PBS) and placed into the 24 well plates, with media for 24 h before any cell seeding (n = 6). Each polymer disk was seeded with 1 × 10^5^ cells/cm^2^ with 1 ml of cell culture media.

##### Cell viability

To assess the cytotoxicity and viability, Alamar blue^TM^ (Life Technologies, UK) was used. Scaffolds were put in 24 well plates and sterilized as explained before. Samples were moved to fresh well plate following 1 h of cell seeding to only account for attached cells on the scaffold. Alamar blue assay was then performed as per manufacturer's instructions. Briefly, after 4 h of incubation with Alamar blue dye, 100 μl of media was place into 96 well plates and fluorescence was measured at excitation and emission wavelength of 530 and 620 nm using Fluoroskan Ascent FL, (Thermo Labsystems, UK) (n = 6). As this assay is non-toxic to cells, the same set of scaffolds was used for further testing by washing them with PBS and replacing with fresh cell culture media.

##### DNA quantification

To assess fibroblast cells proliferation a Fluorescence Hoechst DNA Quantification Kit was utilized to quantify the DNA content on the scaffolds (Sigma, UK). Assay was performed as per manufacturer's instructions. The fluorescence was measured at excitation and emission wavelength of 360 and 460 nm using Fluoroskan Ascent FL (n = 6) (Thermo Labsystems, UK).

##### Extracellular collagen production

The extracellular collagen production by the HDFs was analyzed at days 7 and 14 using the Picco Sirius Red (PSR) method, as described previously.^17^ Dye contains a reagent that specifically binds to collagen. Briefly, cell seeded scaffolds were first fixed in methanol overnight at − 20 °C. After washing with PBS they were staining at room temperature for 4 h with the 0.1% Picro Sirius Red (PSR) solution (Sigma Aldrich, UK). Excess dye was removed by washing 3 times with 0.1% acetic acid. To quantify the amount of collagen produce per sample, PSR stained samples were incubated with 200 μl of 0.1 N sodium hydroxide. Samples were placed on a rocker at room temperature for 1 h before the optical density (OD) was measured at 540 nm with the Anthos 2020 microplate reader (Biochrome Ltd, UK). Standard curve was prepared using various amounts of bovine collagen (1, 5, 10, 20, 30, 40 and 50 μg) as per manufacture's instruction (n = 6), which was later used to quantify the amount of collagen per sample.

##### Fluorescence Labelling for Cell Morphology

To study cell morphology and cell adhesion of the HDFs on the POSS-PCU scaffolds, actin cytoskeleton staining using phalloidin was performed. Firstly, the cell culture media were removed from the 24-wells and samples were washed with PBS three times. Cells were fixed with 4% (w/v) paraformaldehyde in PBS pre-warmed at 37 °C and left for 10-15 min. Cell permeability was improved by incubating samples with 0.1% Triton-X100 for 5 min. Actin cytoskeleton was stained by incubating samples with Rhodamine–Phalloidin dye (1:40) in PBS, for 40 min. Cells were then washed three times with PBS and incubated with DAPI solution to stain the nuclei. The samples were then mounted on glass slide using prolong gold (Antifading agent) and then visualized under the fluorescent microscope.

##### Histology

The migration of cultured HDFs within the scaffolds was determined using histology. Following 14-day culture, the polymer constructs were harvested and fixed with 10% formalin for 24 h at 4 °C. Following dehydration through a series of graded ethanol solutions, samples were paraffin embedded and horizontally cross sectioned (2-4 μm) from the middle of the scaffolds. Samples were stained with hematoxylin and eosin (H&E) staining (n = 3) to analyze cellular ingrowth.

##### Statistical analysis

Statistical analysis of the results was performed using Prism software. Statistical significance was calculated by using two-way ANOVA coupled with Mann–Whitney test, wherever specified in the text. *P* < 0.05 was considered statistically significant.

## Results

### Material characterization

#### X-ray photoelectron spectroscopy (XPS) analysis

**Figure 1:**
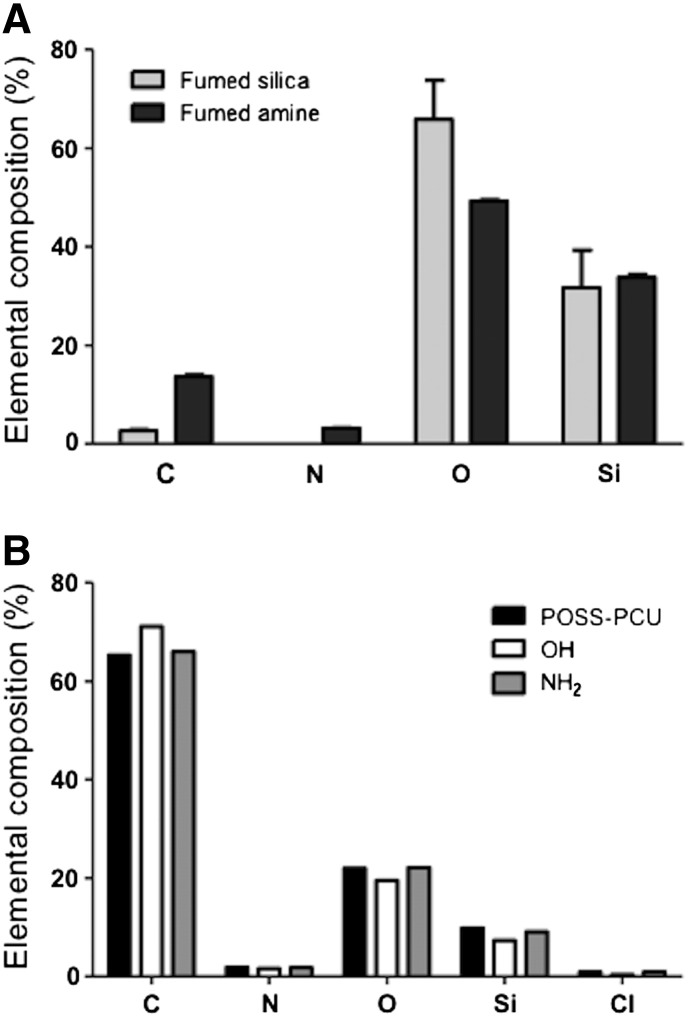
**(A)** Elemental composition of fumed silica and fumed silica amine nanoparticles prior to dispersion within POSS-PCU scaffolds. **(B)** Elemental composition of OH and NH_2_ scaffolds. Key: NH_2_: POSS-PCU modified with amine nanoparticles; OH: POSS-PCU modified with fumed silica nanoparticles; POSS-PCU: unmodified scaffolds.

XPS analysis was performed to investigate changes in the scaffold surface chemistry. Functionalization of fume silica particles with the amine group via silanization was confirmed by the presence of nitrogen (Figure 1, *A*), which was absent on the fumed silica particles. Once modification of fume silica with amine group was confirmed, both fume silica and fumed amine were mixed with POSS-PCU to manufacture the porous scaffolds, which were termed as ‘OH’ and ‘NH_2_’ respectively, based on the type of silica used. The XPS analysis of scaffolds modified by mixing fumed silica (OH) and fumed amine (NH_2_) showed minimal changes in surface chemistry (Figure 1, *B*). Survey scans are presented in supplementary information (Supplementary Figure S3-5). The N, O and Si % remained the same for all the samples suggesting the non-availability of functionalized silica on the surface of scaffolds. Except for FS groups, a higher amount of % C compared to POSS-PCU and NH_2_ was observed. However, it is likely that this increase could be attributed to carbon contamination during processing of samples. Thus, XPS analysis suggests that the surface chemistry remains unchanged after the addition of functionalized silica nano-fillers to POSS-PCU.

#### Atomic force microscopy (AFM)

**Figure 2:**
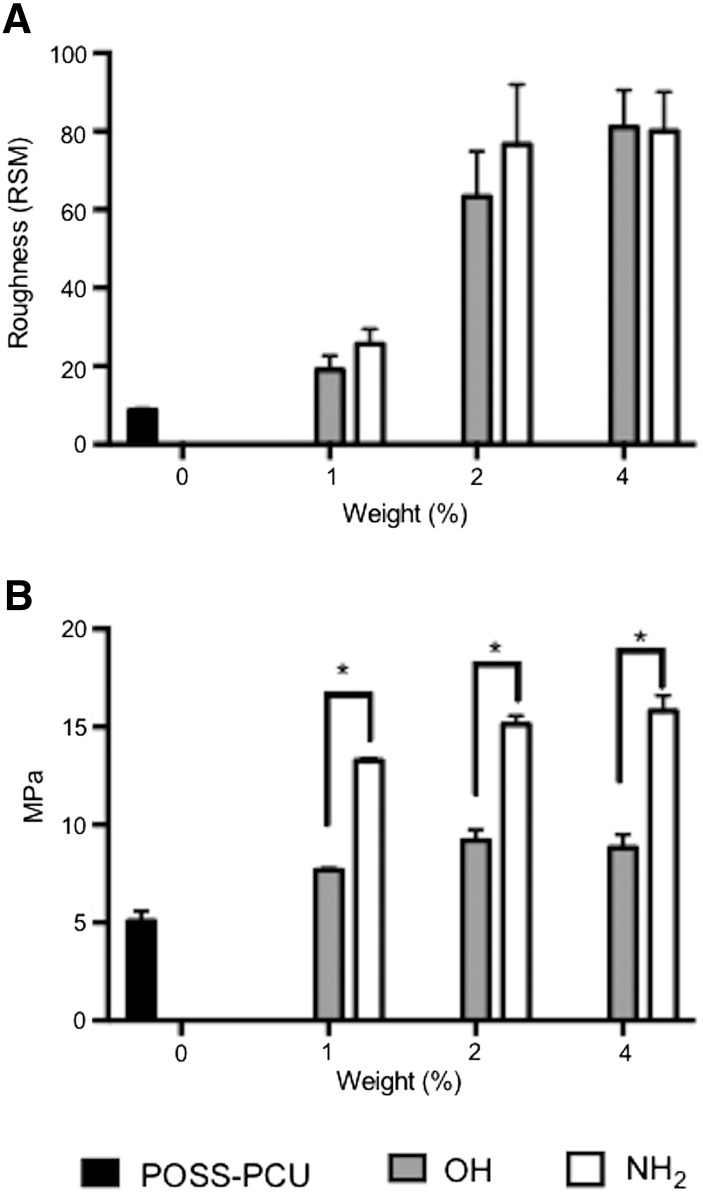
**(A)** Surface roughness measured by atomic force microscopy (AFM) (n = 3). There was no significant difference in roughness between NH_2_ and OH scaffolds using 1, 2 or 4% filler. **(B)** Bulk mechanical stiffness measured by Tensile Young's Elastic Modulus (n = 6). NH_2_ scaffolds were significantly stiffer in tension than OH scaffolds using 1, 2 or 4% filler (*P* < 0.001). Key: NH_2_: POSS-PCU modified with amine nanoparticles; OH: POSS-PCU modified with fumed silica nanoparticles; POSS-PCU: unmodified scaffolds.

AFM was used to assess the nanotopography induced by the addition of the OH and NH_2_ to POSS-PCU nanocomposite polymer (Figure 2, *A*, Supplementary S6). Unmodified POSS-PCU showed an RMS roughness of 9 ± 1 nm. After the addition of 1% of fume silicas (OH and NH_2_) the roughness increased significantly, compared to unmodified POSS-PCU (*P* < 0.01). There was also no significant difference between OH and NH_2_ scaffolds above 1% of silica being added to the scaffolds.

#### Mechanical properties

After the addition of the two different types of silica fillers, the bulk elastic modulus of POSS-PCU scaffolds increased significantly compared to unmodified POSS-PCU (*P* < 0.01). It was observed that the addition of 1% of either type of silica resulted in a significant increase in the bulk elastic modulus (*P* < 0.01). However, above the addition of 2% of both types of nano-silica fillers the Young's elastic modulus remained unchanged (Figure 2, *B*). When OH and NH_2_ were compared for their effect on the mechanical properties of scaffolds, scaffolds with NH_2_ showed a two-fold increased elastic modulus compared to OH incorporated scaffolds.

#### Scanning electron microscopy (SEM)

To assess if the addition of the nano-silica fillers affected the structure of POSS-PCU, SEM was used to examine the pore size and porosity. After modification with fumed silica nanoparticles OH-4% and NH_2_-4% showed no difference in the structure including the pore size and surface of the nanocomposite material compared to unmodified POSS-PCU as shown by SEM (Supplementary Figure 1). TEM analysis (Supplementary S7) demonstrates an average size for both fumed silicas as between 25 and 30 nm.

### Cell culture

To assess the effect of modified scaffolds with functional silica nanofillers on cell behavior, an *in vitro* study was conducted with human dermal fibroblast (HDF) cells. Cellular function as a result of modification of scaffolds was examined by cell morphology, proliferation and extracellular matrix (ECM) production.

#### Cell adhesion and morphology

**Figure 3:**
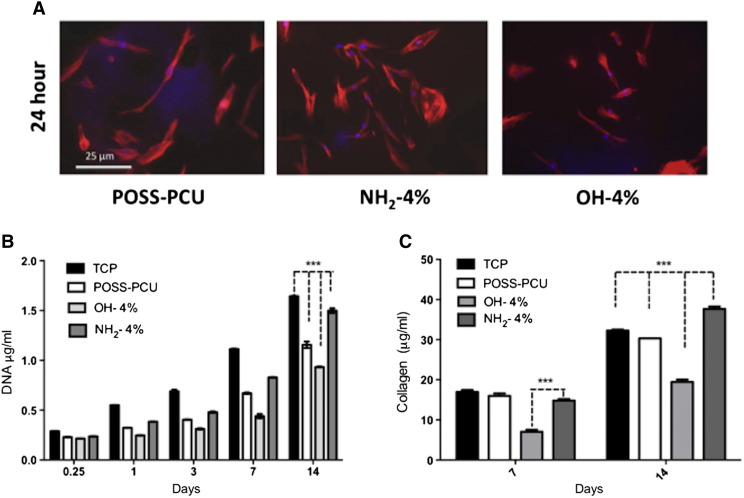
Human dermal fibroblast (HDFs) behavior on the modified scaffolds. **(A)** Immunocytochemistry illustrating the cell morphology of the human dermal fibroblasts adhering to the POSS-PCU scaffolds at 24 h. **(B)** Cell proliferation over 14 days on the POSS-PCU scaffolds. HDFs showed significantly greater proliferation on NH_2_ modified surfaces than OH surfaces after 14 days(*P* < 0.001). **(C)** Extracellular collagen production by human dermal fibroblasts on days 7 and 14. HDFs showed significantly more collagen on NH_2_ modified surfaces than OH surfaces at 7 and 14 days (*P* < 0.001). Key: NH_2_: POSS-PCU modified with amine nanoparticles; OH: POSS-PCU modified with fumed silica nanoparticles; POSS-PCU: unmodified scaffolds.

Cell adhesion and morphology were assessed by staining the cell's cytoskeleton of F-Actin using Phallodin staining. Figure 3, *A* shows HDFs cell typical morphology on a variety of scaffolds. After 24 h of seeding, the cells showed a spread morphology on all scaffolds. This is further quantified by DNA assay as shown in Figure 3, *B*.

### Cell viability and proliferation

Alamar blue assay and total DNA assay were used to assess metabolic activity and cell growth respectively. HDFs showed similar behavior in terms of their metabolic activity and growth when seeded on the modified and unmodified scaffolds of varying nanofiller concentrations (Supplementary Figure 2). Figure 3, *B* shows the effect of 4% nanofiller incorporated scaffolds on cell numbers, where significant increase (*P* < 0.001) in the viability and proliferation was observed on NH_2_ modified scaffold compared to OH and unmodified POSS-PCU scaffolds over a period of 14 days of cell culture. Tissue culture plastic (TCP) was used a positive control, which showed higher cellular growth compared to all other scaffolds. The OH modified scaffolds showed the lowest cell growth compared to NH_2_ and unmodified POSS-PCU scaffolds.

### Extracellular collagen production

**Figure 4:**
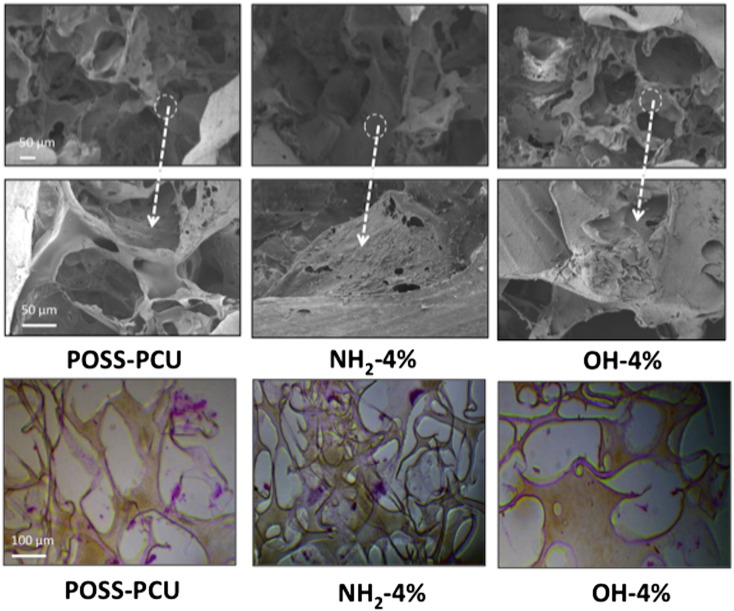
**(A)** Scanning electron microscopy (SEM) images of the scaffolds after 14 days of human dermal fibroblast cell seeding (n = 3). **(B)** Fibroblast migration of the POSS-PCU scaffolds at 14 days using H&E staining (n = 3). Key: NH_2_: POSS-PCU modified with amine nanoparticles; OH: POSS-PCU modified with fumed silica nanoparticles; POSS-PCU: unmodified scaffolds.

Collagen production was used to assess the HDF ability to lay down ECM. The cells showed a significantly lower (*P* < 0.001) amount of collagen deposition on OH modified samples compared to NH_2_ and POSS-PCU at day 7 (Figure 3, *C*). TCP was used as a positive control. After day 14, collagen production on OH modified scaffold increased, but continued to be lower than the other two samples. Collagen production significantly increased (*P* < 0.001) on NH_2_ scaffolds compared to all other scaffolds. Using SEM analysis at 14 days, it was observed that HDFs on NH_2_ scaffolds were able to lay down ECM to a greater degree than on POSS-PCU and OH scaffolds (Figure 4).

### Cell migration

The scaffolds used during this study were porous. Thus, cell migration into the porous structure was investigated by histological analysis. Samples were section through the middle of the scaffold after 14 days of cell seeding and stained for H&E analysis as shown in Figure 4, *B*. A greater number of cells was found to be present in the middle of NH_2_ compared to OH and POSS-PCU scaffolds.

## Discussion

Over recent years, researchers have explored the ways of modifying properties of biomaterials to manipulate cellular behavior.^17^, ^18^, ^19^, ^20^, ^21^, ^22^, ^23^, ^24^ Current study investigates a simple and effective method for the production of mechanically-responsive scaffolds using silica nano-fillers and their effect on cellular functions. The scaffolds are made from a biocompatible polymer called POSS-PCU. In order to change mechanical properties of scaffolds, two types of silica with OH and NH_2_ functional group are mixed with the polymer to produce scaffolds. Fume silica itself has hydroxyl (OH) functionality; NH_2_ functional group was introduce on to fume silica using the classical amino-silanization process. Functionalization of silica with NH_2_ functionality was confirmed by XPS analysis. A increase in the N% was only observed for amino silane modified silica (Figure 1, *A*) confirming the amine functionalization of fume silica. An increase in C% and decrease in O% from fume silica after amino silanization further confirmed chemical coupling of silane molecules. These two functional silica nanoparticles were then mixed with POSS-PCU polymer at various concentrations to study their effects on bulk mechanical properties, roughness and surface chemistry.

When scaffolds were analyzed for a change in the surface chemistry after functional nano-silica fillers were mixed with POSS-PCU, no significant changes in surface chemistry were observed via XPS analysis. XPS is a well-known surface analysis technique, widely used for quantitative assessment of surface chemical analysis. The elemental analysis of scaffolds pre- and post-silica incorporation is presented in Figure 1, *B*. The presence of C, N and O along with Si was used to identify POSS-PCU background spectra, part of O and Si signal is related to POSS nanocage and part of C, N and O is from the PCU component. The increase in either O% or N% or Si% was used to detect either OH and NH_2_ functionalized silicas. Since all the samples showed the same level of elemental composition, it can be confirmed that the surface chemistry remains unaffected by the addition of OH or NH_2_ functionalized silicas. The XPS probing depth is around 10 nm, meaning that if a continuous film of this thickness was formed, the scaffolds would totally attenuate the Si signal from the substrate. The absence of the fume silica specific signal or significant change in the N% or O% compared to unmodified POSS-PCU suggested that silica particles are below this depth at interface of the scaffold. It is possible that, when particles are added to polymeric phase and cured by solvent casting method, particles sink in bulk phase of the polymer and beyond the depth resolution of XPS. However, the nanoparticles remained close enough to bring changes to surface roughness of the scaffolds as shown by AFM analysis (Figure 1, *C*), where surface roughness increased from 20 nm to 80 nm following incorporation of 1-4% nano-silicas. No significant difference was observed in surface roughness of the scaffolds when using either type of fumed silica.

**Figure 5:**
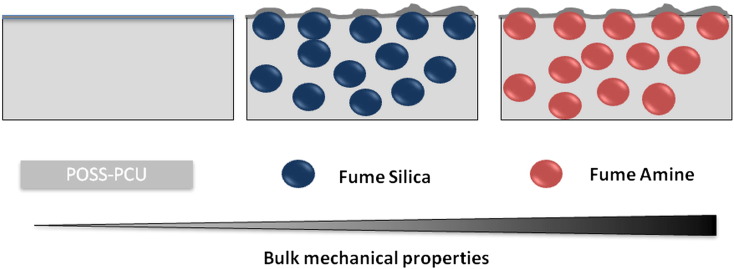
Schematic diagram to illustrate changes to POSS-PCU scaffolds after the incorporation of nanofillers. Key: NH_2_: POSS-PCU modified with amine nanoparticles; OH: POSS-PCU modified with fumed silica nanoparticles; POSS-PCU: unmodified scaffolds.

Thus, to conclude from surface and bulk mechanical analysis, though bulk mechanical properties were changed with the use of two different silica fillers, no significant changes were observed in the surface chemistry. Silica particles probably remained close to the surface of scaffolds producing a nano-roughness at the interface. Schematic representation of this hypothesis is presented in Figure 5. However, this hypothesis will need further evaluation in terms of specific interactions of functional silicas with POSS-PCU scaffold, which will be part of our future investigation.

The most interesting finding in this study was investigating the mechanical properties of scaffolds after the incorporation of these nano-silica fillers. The mechanical stiffness increased following incorporation of both OH and NH_2_ functionalized silica fillers. However, there was a significantly higher bulk Young's modulus (*P* < 0.01) with NH_2_ functionalized silica compared to OH functionalized and unmodified scaffolds. Incorporation of as low as 1% of silica nanoparticles caused significant changes in mechanical properties for both types of scaffolds. It is already well known that fumed silica nanoparticles act as a filler to materials and hence contribute toward the strength of materials.^25^ Chrissafis et al^26^ found that incorporating fumed silica nanoparticles substantially increased tensile strength and Young's modulus of poly(vinyl pyrrolidone) (PVP), chitosan (Chi), or poly(vinyl alcohol) (PVA) based hydrogels. However, to the best of our knowledge this is the first example where functional silicas were used into polymeric scaffolds to influence mechanical properties.

To evaluate the effect of these modified scaffolds on cellular functions, human dermal fibroblasts (HDFs) were cultured on the scaffolds and their adhesion, spreading and long term survival were analyzed using a variety of assays. From Figure 2, *A*, it is clear that cells on unmodified POSS-PCU and NH_2_ scaffolds showed a spread morphology compared to OH modified scaffolds. From Figure 2, *A* it is also clear that a lower number of cells attached on OH functional scaffolds than NH_2_ and unmodified scaffolds, which is later quantified using DNA assay (Figure 2, *B*). When cells were cultured over a 14 day period, the cell growth profile was found to be in the order of OH < POSS-PCU < NH_2._ Nano-silica fillers with NH_2_ showed significantly higher collagen production compared to other scaffolds, which could be attributed to higher cell growth on these scaffolds. Increase in fume silica content from 1 to 4 wt% did not affect cell growth, suggesting that 1 wt% silica addition was enough to either promote or discourage cell growth as seen from Supplementary Figure S1.

Mechanoregulation of cell fate has been extensively studied, with several reports demonstrating that substrate stiffness can also be used to direct adult stem cell behavior.^23^ It is now increasingly evident that bulk properties of materials play a pivotal role in cellular function. Several studies have compared soft and hard materials, observing differences in cellular adhesion, morphology and differentiation depending on the mechanical substrate stiffness.^20^, ^21^, ^22^, ^23^ Increased substrate stiffness has shown to promote fibroblast adhesion, proliferation and phenotype in several studies.^4^, ^5^, ^7^ Seo et al^7^ showed that fibroblasts adhered to stiffer polydimethylsiloxane substrates (PDMS) because of the optimal orientation of the cell binding motif of fibronectin. Similar study by Fusco et al^5^ using polyacrylamide and polydimethylsiloxane gels ranging from 3 to 100 kPa and showed greater cell focal adhesion formation on stiffer substrates for fibroblast cells. In this study, NH_2_ scaffolds showed greater cell response than OH scaffolds due to an increased mechanical stiffness. It is probable that the optimal stiffness of the NH_2_ scaffolds has resulted into optimal protein adsorption and conformation as seen in previous studies and enabled enhance cells adhesion, spreading and consequently proliferation with time. However, this can be part of further investigation.

This study provides a simple but efficient method to direct cellular functions by using functional nano-silica fillers. The addition of a small percentage of silica nanofiller brought about significant changes in the bulk properties of the scaffold. These changes in bulk properties can be tailored using different types of silicas, thereby selectively altering the cellular functions. This study paves a way to fabricate composite scaffold structures where appropriate cell response can be achieved by changing the material's mechanical properties.

## Appendix A Supplementary data

Figure S1. Scanning electron microscopy images of the scaffolds after modification with the fumed silica nanoparticles and fumed silica nanoparticles functionalized with amine nanoparticles (n = 3). Key; NH_2_: POSS-PCU modified with amine nanoparticles OH: POSS-PCU modified with fumed silica nanopartilces POSS-PCU: Unmodified scaffolds. Figure S2. Human dermal fibroblast behaviour over 14 days on the modified scaffolds with increasing amounts of silica nanoparticles. (A) Cell viability using alamar blue assay (n = 6). (B) Total DNA assay (n = 6). HDFs showed a similar cell viability and growth on the modified scaffolds over a 14 day period. Key; NH_2_: POSS-PCU modified with amine nanoparticles OH: POSS-PCU modified with fumed silica nanopartilces POSS-PCU: Unmodified scaffolds. Figure S3. X-ray photoelectron spectroscopy (XPS) survey spectra of POSS-PCU. Figure S4. X-ray photoelectron spectroscopy (XPS) survey spectra of POSS-PCU modified with fumed silica nanoparticles. Figure S5. X-ray photoelectron spectroscopy (XPS) survey spectra of POSS-PCU modified with fumed silica nanoparticles functionalized with amine groups (NH_2_). Figure S6. Atomic Force Microscopy Images of the POSS-PCU scaffold and POSS-PCU modified with 4% fumed silica nanoparticles (OH) and 4% fumed silica nanoparticles modified with amine (NH_2_). Figure S7. Transmission Electron Microscopy (TEM) of the fumed silica nanoparticles (OH) and fumed silica nanoparticles modified with amine (NH_2_).
