# Supplementary material for: Development of mechano-responsive polymeric scaffolds using functionalized silica nano-fillers for the control of cellular functions
Source: Nanomedicine. 2016 Aug;12(6):1725–33. doi: 10.1016/j.nano.2016.02.011 (PMC4949378; doi:10.1016/j.nano.2016.02.011)
Supplement: Supplementary file 1 — Figure S1. Scanning electron microscopy images of the scaffolds after modification with the fumed silica nanoparticles and fumed silica nanoparticles functionalized with amine nanoparticles (n = 3). Key; NH2: POSS-PCU modified with amine nanoparticles OH: POSS-PCU modified with fumed silica nanopartilces POSS-PCU: Unmodified scaffolds. Figure S2. Human dermal fibroblast behaviour over 14 days on the modified scaffolds with increasing amounts of silica nanoparticles. (A) Cell viability using alamar blue assay (n = 6). (B) Total DNA assay (n = 6). HDFs showed a similar cell viability and growth on the modified scaffolds over a 14 day period. Key; NH2: POSS-PCU modified with amine nanoparticles OH: POSS-PCU modified with fumed silica nanopartilces POSS-PCU: Unmodified scaffolds. Figure S3. X-ray photoelectron spectroscopy (XPS) survey spectra of POSS-PCU. Figure S4. X-ray photoelectron spectroscopy (XPS) survey spectra of POSS-PCU modified with fumed silica nanoparticles. Figure S5. X-ray photoelectron spectroscopy (XPS) survey spectra of POSS-PCU modified with fumed silica nanoparticles functionalized with amine groups (NH2). Figure S6. Atomic Force Microscopy Images of the POSS-PCU scaffold and POSS-PCU modified with 4% fumed silica nanoparticles (OH) and 4% fumed silica nanoparticles modified with amine (NH2). Figure S7. Transmission Electron Microscopy (TEM) of the fumed silica nanoparticles (OH) and fumed silica nanoparticles modified with amine (NH2). [file mmc1.doc]

**Supplementary data**

**Title: Development of mechano-responsive polymeric scaffolds using functional silica nano fillers for control cellular functions**

Griffin M,^1^ Nayyer L.^1^, Butler P.E.^1,2^, Palgrave R.G.^3^, Seifalian A.M., Kalaskar D. M.*^1^

*^1^UCL Centre for Nanotechnology and Regenerative Medicine, Division of Surgery & Interventional Science, University College London, London, United Kingdom*

*^2^Royal Free London NHS Foundation Trust Hospital, London, United Kingdom*

*^3^Department of Chemistry, University College London, 20 Gordon Street, London, WC1H 0AJ.*


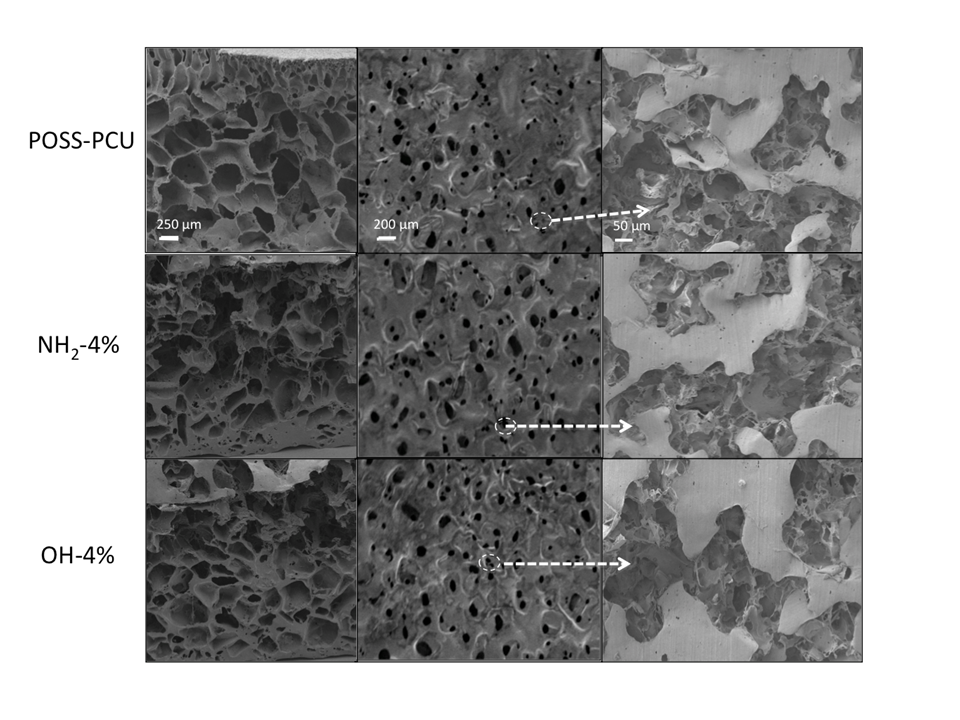


**Figure S1. Scanning electron microscopy images of the scaffolds after modification with the fumed silica nanoparticles and fumed silica nanoparticles functionalized with amine nanoparticles (n=3). Key; NH_2_: POSS-PCU modified with amine nanoparticles OH: POSS-PCU modified with fumed silica nanopartilces POSS-PCU: Unmodified scaffolds.**

**
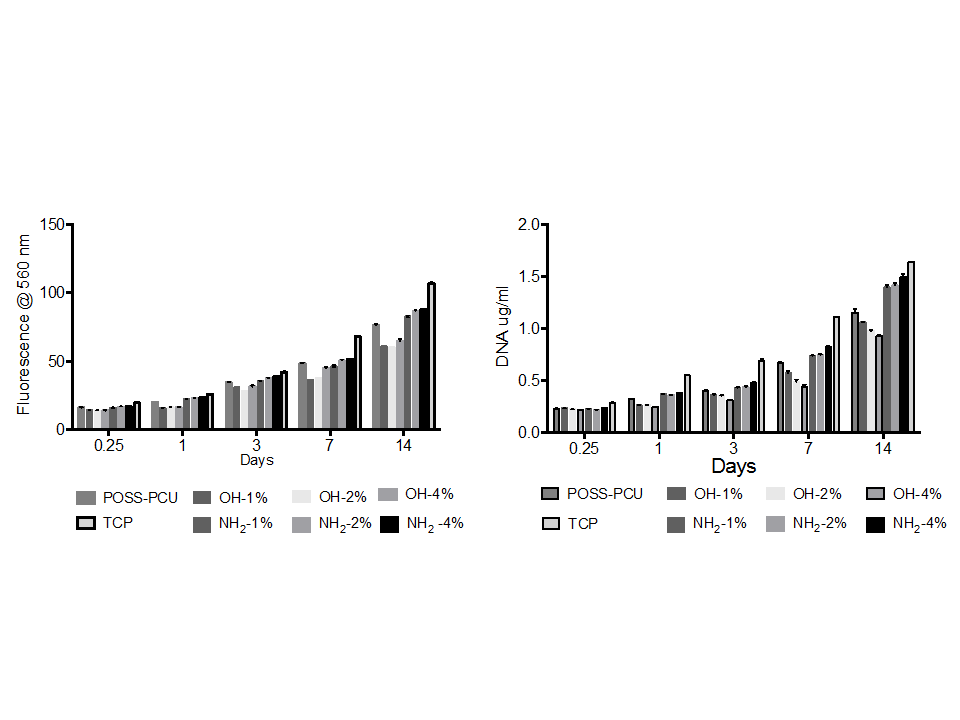
**

**Figure S2. Human dermal fibroblast behaviour over 14 days on the modified scaffolds with increasing amounts of silica nanoparticles. (A) Cell viability using alamar blue assay (n = 6). (B) Total DNA assay (n = 6). HDFs showed a similar cell viability and growth on the modified scaffolds over a 14 day period. Key; NH_2_: POSS-PCU modified with amine nanoparticles OH: POSS-PCU modified with fumed silica nanopartilces POSS-PCU: Unmodified scaffolds.**

**
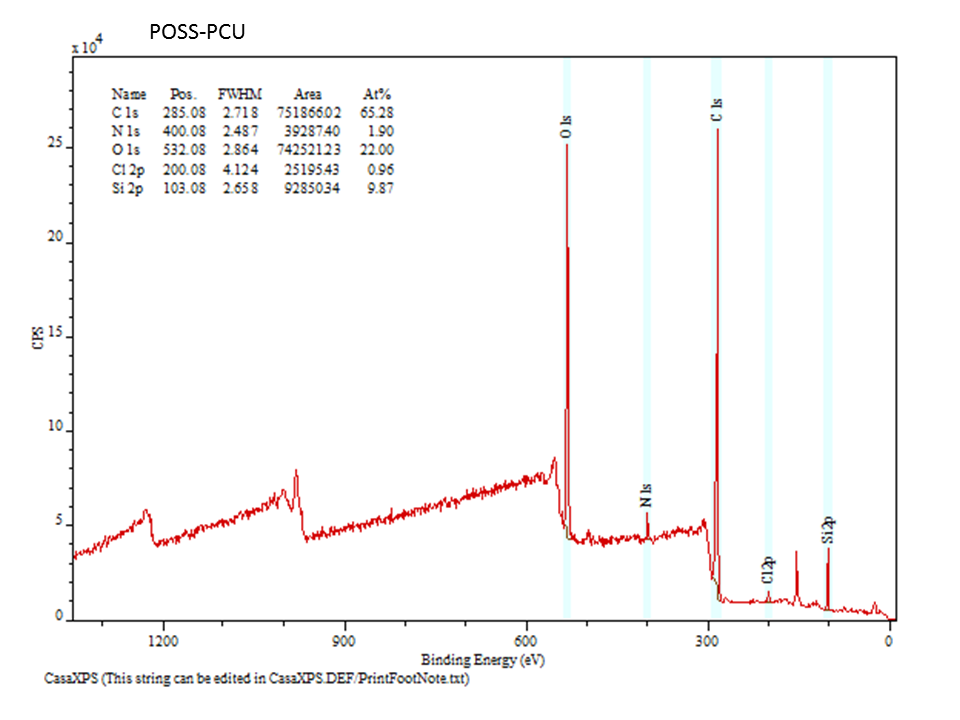
**

**Figure S3. X-ray** [**photoelectron**](http://en.wikipedia.org/wiki/Photoelectron) [**spectroscopy**](http://en.wikipedia.org/wiki/Spectroscopy) **(XPS) survey spectra of POSS-PCU.**

**
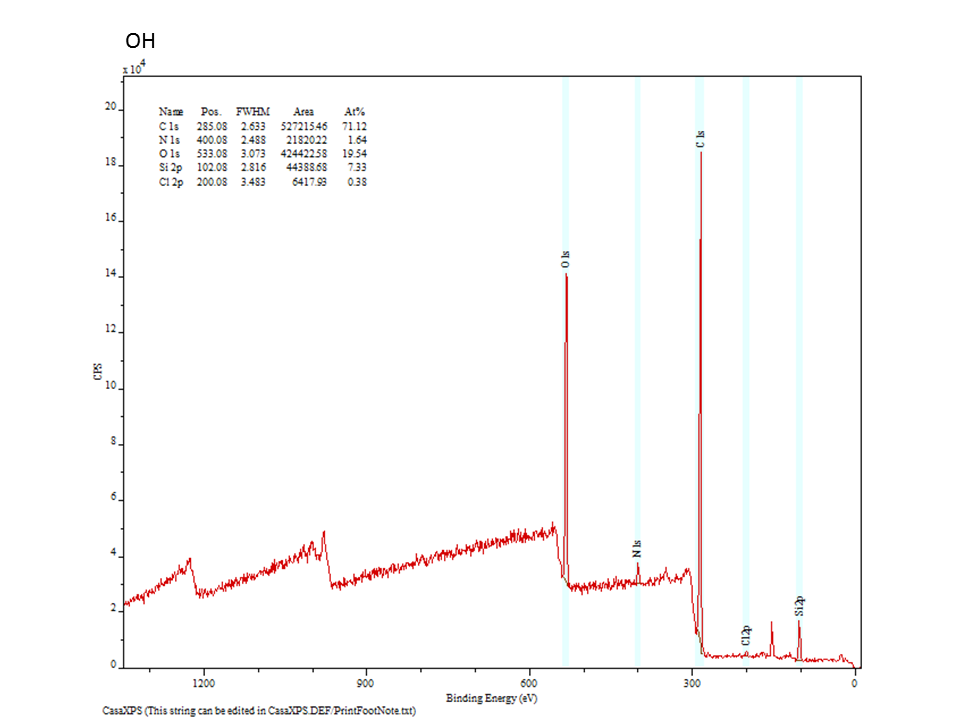
**

**Figure S4. X-ray** [**photoelectron**](http://en.wikipedia.org/wiki/Photoelectron) [**spectroscopy**](http://en.wikipedia.org/wiki/Spectroscopy) **(XPS) survey spectra of POSS-PCU modified with fumed silica nanoparticles .**

**
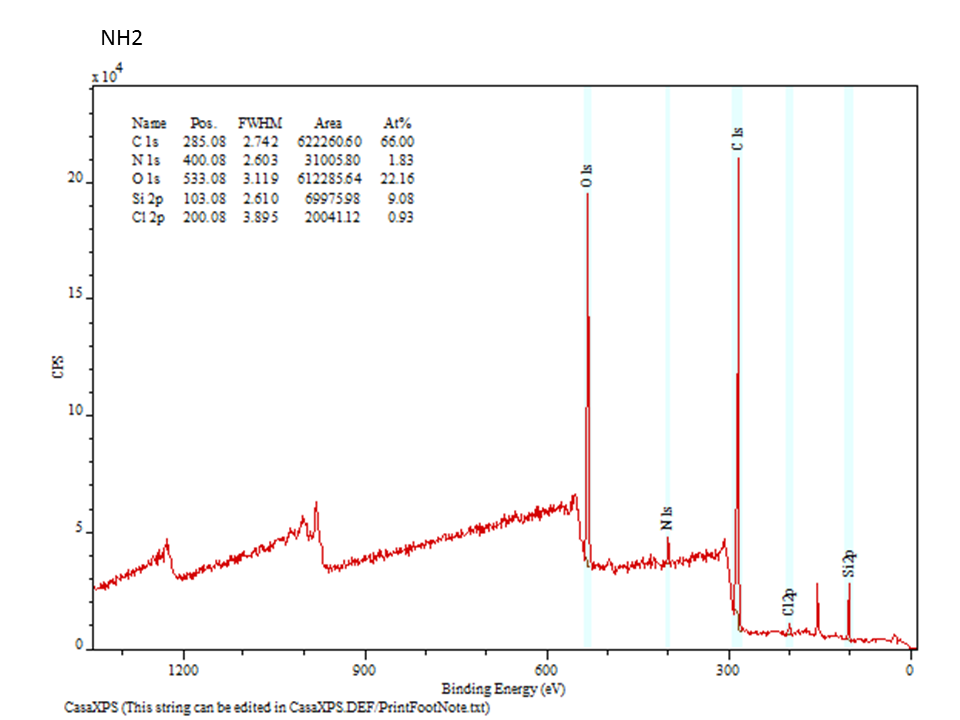
**

**Figure S5. X-ray** [**photoelectron**](http://en.wikipedia.org/wiki/Photoelectron) [**spectroscopy**](http://en.wikipedia.org/wiki/Spectroscopy) **(XPS) survey spectra of POSS-PCU modified with fumed silica nanoparticles functionalized with amine groups (NH_2_) .**


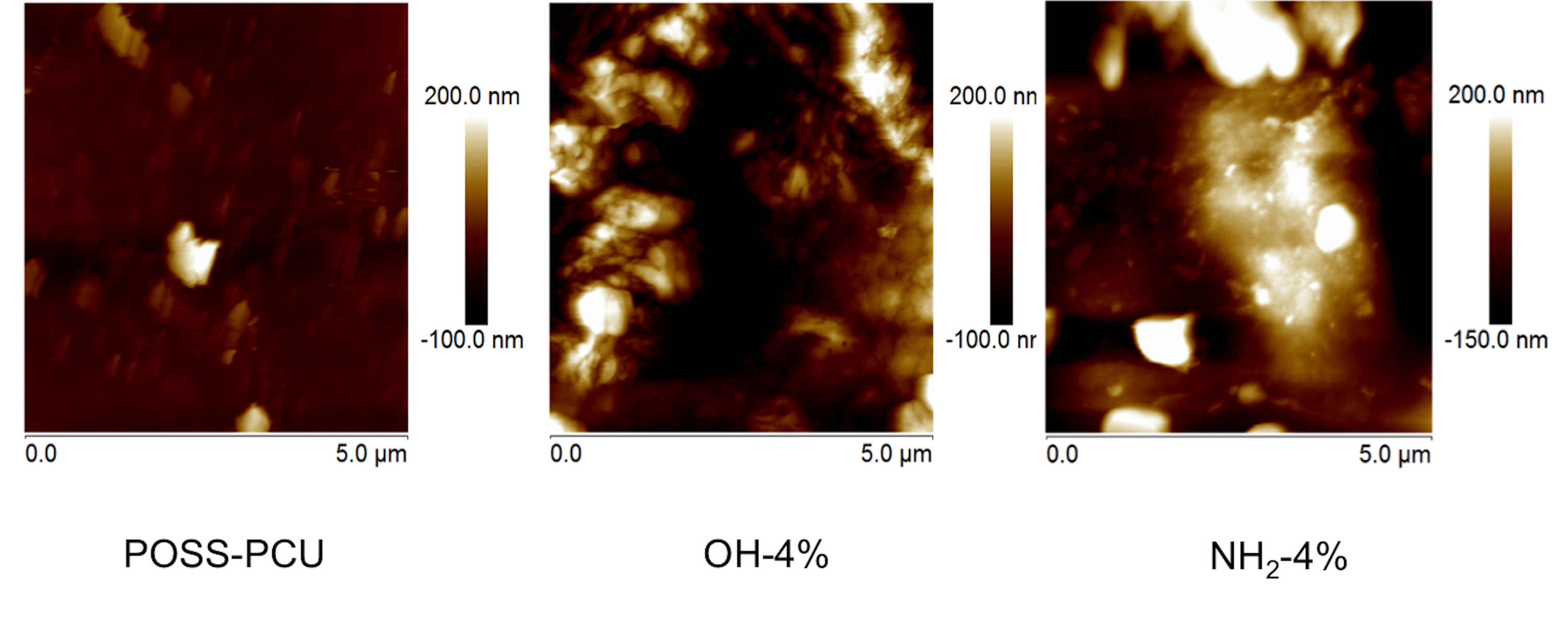


**Figure S6 Atomic Force Microscopy Images of the POSS-PCU scaffold and POSS-PCU modified with 4% fumed silica nanoparticles (OH) and 4% fumed silica nanoparticles modified with amine (NH_2_).**


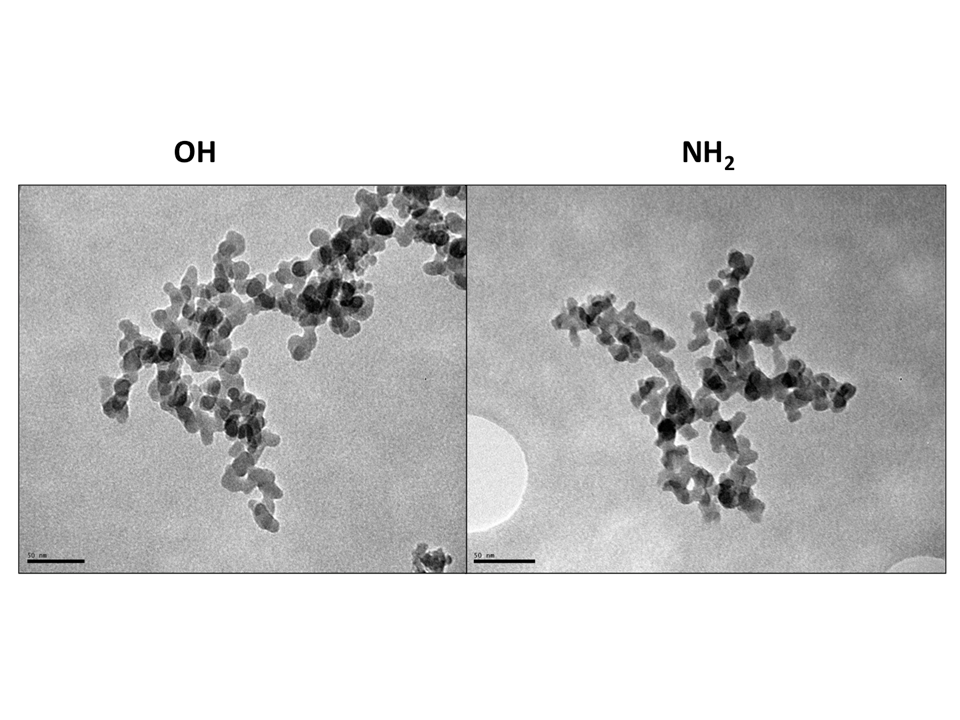


**Figure S7. Transmission Electron Microscopy (TEM) of the fumed silica nanoparticles (OH) and fumed silica nanoparticles modified with amine (NH_2_).**
